# Cyclospora Cayetanensis—Major Outbreaks from Ready to Eat Fresh Fruits and Vegetables

**DOI:** 10.3390/foods9111703

**Published:** 2020-11-20

**Authors:** Agni Hadjilouka, Dimitris Tsaltas

**Affiliations:** 1 EMBIO Diagnostics LTD., Athalassas 8b, 2018 Nicosia, Cyprus; agnixatz@gmail.com; 2 Department of Agricultural Sciences, Biotechnology and Food Science, Cyprus University of Technology, Archbishop Kyprianos 30, 3036 Limassol, Cyprus

**Keywords:** *Cyclospora cayetanensis*, major outbreaks, fresh produce

## Abstract

*Cyclospora cayetanensis* is a coccidian protozoan that causes cyclosporiasis, a severe gastroenteric disease, especially for immunocompromised patients, children, and the elderly. The parasite is considered as an emerging organism and a major contributor of gastroenteritis worldwide. Although the global prevalence of cyclosporiasis morbidity and mortality has not been assessed, global concern has arisen since diarrheal illness and gastroenteritis significantly affect both developing countries and industrialized nations. In the last two decades, an increasing number of foodborne outbreaks has been associated with the consumption of fresh produce that is difficult to clean thoroughly and is consumed without processing. Investigations of these outbreaks have revealed the necessity to increase the awareness in clinicians of this infection, since this protozoan is often ignored by surveillance systems, and to establish control measures to reduce contamination of fresh produce. In this review, the major cyclosporiasis outbreaks linked to the consumption of ready to eat fresh fruits and vegetables are presented.

## 1. Introduction

Diarrhea is one of the leading causes of mortality worldwide. In 2016, it was responsible for the death of more than 1.6 million people, with 90% of the deaths being reported in South Asia and sub-Saharan Africa [1]. It is also estimated that diarrhea is the second cause of death in children under five, killing nearly 525,000 every year [2]. Enteric protozoan parasites are among the leading causes of human diarrhea disease [3]. *C. cayetanensis* is one of these protozoan parasites that is considered as an emerging organism able to cause cyclosporiasis, a severe gastro-enteric disease especially for immunocompromised patients, children, and elderly.

*Cyclospora* is an apicomplexan, cyst-forming coccidian protozoan that belongs to subphylum Apicomplexa, subclass Coccidiasina, and family Eimeriidae [4]. Currently, nineteen *Cyclospora* species have been reported to be causative agents of disease in various animal species [5] and only one species, *C. cayetanensis*, is known to be associated with syndromes of acute and chronic diarrhea in humans. It is endemic in tropical and subtropical areas, but it has been identified as the causative agent of several outbreaks worldwide in the last two decades and it is also considered as a significant contributor to global gastroenteritis spread [6]. Most of the cyclosporiasis cases reported in non-endemic areas have been linked either with travelers returning from endemic areas or with imported foods from developing countries. It is however noteworthy that there is a significant number of reported cases in non-endemic areas, as the risk of exposure to uncommon diseases has been increased. This increase has mainly occurred because of the augmentation of international travel, the globalization of food supply and change in consumers’ dietary habits [4]. *Cyclospora* is highly resistant to disinfectants used in the food industry [4], while globalization of food supply disseminates pathogenic organisms over wide geographical areas introducing important hazards. Besides, thanks to the increasing popularity of fresh fruits and vegetables—due to consumers’ tendency to eat healthier—produce have become important vehicles in food-borne illness statistics [7].

## 2. Historic Taxonomy and Molecular Characteristics

The genus *Cyclospora* was first described in Old World moles (Talpinae) and subsequently in reptiles, birds, and myriapods [8,9]. The first documented human cases were diagnosed in 1977 and 1978 and reported by Ashford in 1979 [10], who isolated the organism from the stools of three patients in Papua New Guinea and correctly concluded that the organism was a coccidian parasite. However, because of its morphology and his uncertainty about the number of the sporozoites in the sporocysts, he wrongly presumed that the organism was a species of *Isospora* [10].

In 1986 Soave et al. [11] reported a new enteric pathogen that was the causative agent of a flu-like illness in four travelers to Haiti and Mexico, while in 1989 Naranjo et al. [12] described a ‘*Cryptosporidium muris*-like object’ reporting that it was probably an unidentified flagellate. In following reports, the organism was described as a cyanobacterium-like body, coccidian-like body (CLB), blue-green algae, or large *Cryptosporidium* [13,14,15,16]. Eventually, in 1993 Ortega et al. [17] classified this new organism in the genus *Cyclospora* and in 1994 they proposed the name *C. cayetanensis* for the new human parasite (derived from their university —Universidad Peruana Cayetano Heredia) [18]. Since then, many studies have been performed to identify the biological and epidemiological characteristics of this organism.

Phylogenetic analysis of the *C. cayetanensis* small subunit rRNA (SSU rRNA) gene sequences and mitochondrial genome have demonstrated that this organism is closely related to the *Eimeria* genus [19,20], while phylogenetic analysis based on the 18S rDNA sequences has indicated that this relation can even be compared to that existing within *Eimeria* species [21]. At the same time, cluster analysis of apicoplast (non-photosynthetic plastids) genomes originating from different Apicomplexan parasites have revealed a strong relation among *C. cayetanensis*, *Eimeria*, and *Toxoplasma,* and a common evolutionary history [22].

Lack of geographic segregation and existence of genetically homogeneous *C. cayetanensis* parasites has also been demonstrated [23,24]. In 2013, Sulaiman et al. reported a lack of genetic polymorphism at the regions of 70kDa heat shock protein (HSP70) locus, while a year later a study revealed minimal genetic diversity among 17 human *C. cayetanensis* isolates based on their 18S ribosomal RNA gene [24]. In 2016, Cinar et al. [22] performed comparative analysis of 11 *C. cayetanensis* apicoplast genomes originating from different geographical areas and reported high conservation with only a few polymorphisms. Nevertheless, it has not been indicated yet whether all human *Cyclospora* isolates belong to the same species, or that species that are disease agents in low primates cannot also infect humans [6].Studies have reported that *C. cayetanensis* was not detected in primates, suggesting that some primates are unlikely to be reservoirs for the human pathogen [21,25,26]. Moreover, the strong relationship that characterizes *Cyclospora* and *Eimeria* suggests that *Cyclospora* is probably a host-specific pathogen [27]. However, the pathogen genome’s further characterization is necessary to improve knowledge of this organism regarding its taxonomic position, as well as to understand its transmission dynamics and pathogenic potential.

## 3. Infection

*Cyclospora* is a fecal-oral pathogen with spherical oocysts that measure 8 to 10 μm in diameter. Its oocysts are unsporulated when excreted and require a few days or weeks to become sporulated and infectious, depending upon the environmental conditions [28]. Human infection occurs via produce consumption or contact with fecally contaminated water, sewage, and soil [6]. Person-to-person transmission is unlikely because of the required sporulation period outside of the host [28], as is transmission via ingestion of newly contaminated food or water [29]. The infectious dose is considered to be relatively low and the incubation period ranges from 1 to 11 days [28].

The organism infects the upper intestine and causes watery diarrhea, anorexia, nausea, abdominal cramps, fatigue, body aches, low-grade fever and weight loss [30]. In general, the infection severity depends mainly on the immune system of the host. For most people, cyclosporiasis is treatable and not life-threatening, while asymptomatic infections have also been observed [31]. However, symptoms in immunocompromised patients are more severe; significant weight loss, intestinal injuries and prolonged diarrheas have been reported [32,33,34]. As in immunocompromised patients, children and the elderly may also develop an acute illness. In endemic areas, the disease is more severe in young children, but it becomes milder as they age, probably due to their frequent exposure to the parasite. This results in shorter and asymptomatic infections and finally in the absence of symptomatic infections in adults. Contrariwise, in non-endemic areas, illness is almost always symptomatic [3,6]. Τhe illness may last from a few days to a month or longer and the symptoms may relapse one or more times.

The clinical diagnosis of the disease can be performed either by microscopical observations of oocysts isolated by patient’s fecal samples (e.g., light microscopy/UV epifluorescence microscopy) or by several molecular tools that are being continuously developed (e.g., PCR, RT-qPCR and MLST) [19,35,36,37]. Microscopical observations are simple methods based on oocysts’ morphology and/or autofluorescence; however, they often lead to false positive or false negative results [38]. On the other hand, methods based on molecular techniques can provide reliable results that could be used for robust detection of the source of disease and discrimination of the different clusters.

The recommended treatment for cyclosporiasis is a combination of two antibiotics, trimethoprim, and sulfamethoxazole, (TMP/SMX), also known as Bactrim, Septra, and Cotrim. TMP/SMX is considered as an effective treatment, however, in case of intolerance to sulfonamide drugs, ciprofloxacin or nitazoxanide are recommended [38]. Drug administration is necessary for people with a weakened immune system, while most people who have healthy immune systems can recover without it [29].

## 4. Cyclosporiasis Outbreaks

Cyclosporiasis outbreaks have been reported since the mid-1990s in USA, Canada, Europe, and Australia where they are usually linked to consumption of fresh produce, such as raspberries, basil, mesclun, lettuce, snow peas, cilantro, and green onions (Table 1). To date, no commercially frozen produce, cooked foods or peeled fruit have been associated with cyclosporiasis infection. Major outbreaks linked to fresh produce consumption from 1995 to 2020 are summarized here.

### 4.1. Raspberries

- United States and Canada, 1996

The outbreak that established *C. cayetanensis* as a foodborne pathogen was reported in the spring of 1996. From May to August, a total of 1465 cases of cyclosporiasis were identified in the United States (20 states and the District of Columbia) and in Canada (Ontario and Quebec) and reported to the Centers of Disease Control and Prevention (CDC) and to Health Canada. Of these cases, 725 were cluster-associated and 740 were sporadic, while 978 cases were confirmed by laboratories. Seven hundred and seventy-two patients were female, 41 were children and 3 people were known to be infected with HIV. Twenty-two people were hospitalized, but no deaths were reported. Based on the people with available clinical information, sporadic patients’ ages ranged from 1 to 92 years with a median age of 49 years [39].

Case-control studies conducted in New York City, NY, USA (New Jersey Department of Health and Senior Services) and Florida demonstrated that consumption of raspberries was significantly associated with the illness. Hence, the Food and Drug Administration (FDA), CDC, and public health authorities of the United States and Canada started an investigation to trace the source of raspberries involved in the outbreak. Traceback of the products showed that the raspberries were produced in Guatemala. However, by the time Guatemalan raspberries were implicated as the causative agents of the outbreak, the growing season in Guatemala was ending and exports had been noticeably decreased [40]. During the next fall and winter, a major export season for Guatemalan raspberries, no outbreaks were reported. Nevertheless, the Guatemalan Berry Commission, in consultation with the FDA and CDC, set up a Hazard Analysis and Critical Control Point (HACCP) system to improve the conditions on individual farms [30].

- United States and Canada, 1997

Despite the control measures implemented by the Guatemalan Berry Commission, another outbreak of cyclosporiasis linked to consumption of Guatemalan raspberries was reported in the United States and Canada in 1997. The onset dates of the illness ranged from 19 March to 3 June and 804 people were infected in total. Approximately 140 laboratory-confirmed and 370 clinically defined cases were recognized in eight states (California, Florida, Maryland, Nebraska, Nevada, New York, Rhode Island, and Texas) and Canada (Ontario). In addition, 224 cases (4 laboratory-confirmed and 220 clinically defined) were reported by people who were on a cruise ship departed from Florida and 70 laboratory-confirmed sporadic cases were reported in the United States and Canada. Investigations indicated that raspberries imported from Guatemala were the vehicle of infection, but the mode of contamination was not determined. The outbreak ended shortly after the government of Guatemala and the Guatemalan Berries Commission voluntarily canceled exports of fresh raspberries to the United States. Nevertheless, apart from raspberries, some cases were linked to mesclun (mixture of baby leaves of lettuce) consumption, even though the source of the implicated mesclun was not determined [41].

- Canada, 1998

Due to the 1996 and 1997 outbreaks that occurred in the USA and Canada, in the spring of 1998 the FDA prohibited the importation of fresh Guatemalan raspberries into the United States. Contrary to this, the Canadian Food Inspection Agency (CFIA) considered Guatemalan raspberries as low risk and continued the import; this decision led in the third consecutive year of cyclosporiasis outbreaks linked to consumption of fresh Guatemalan raspberries.

More accurately, on 2 June a laboratory-confirmed case of cyclosporiasis was reported to Toronto Public Health. The patient had attended a dinner in a hotel in Toronto on 8 May. Subsequently, 6 other people who attended the dinner reported having developed a gastrointestinal infection. Of the 174 people who attended the dinner, 128 were interviewed and 29 of them were infected. The berry garnish with raspberries, blackberries, strawberries and possibly blueberries was initially associated with the illness, but further investigations demonstrated that only raspberries were the causative agent of the infection. In addition to this cluster, twelve more clusters were reported with 163 cases of cyclosporiasis. Raspberries were the only common food in all 13 clusters, while traceback investigations in 8 clusters demonstrated that the raspberries originated in Guatemala [42].

- United States (Pennsylvania), 2000

In 2000, the Philadelphia Department of Public Health in coordination with the CDC performed an epidemiological investigation of a gastrointestinal illness reported on 18 June. The outbreak was associated with 83 attendees of a wedding reception held on 10 June in Philadelphia, Pennsylvania. Of the 83 attendees, 79 guests and members of the wedding were interviewed; 54 of them met the case definition. None of the catering staff reported developing illness during the investigation period; however, on 18 July, the local health department was notified of a female caterer who developed laboratory-confirmed cyclosporiasis eight days after the wedding. She also reported two other catering staff who had developed gastrointestinal illness six days after the reception.

Stool examinations revealed the presence of *Cyclospora* oocysts and food items were examined to determine the source of the infection. Multivariate analysis indicated that the wedding cake, which had a creamy filling with raspberries, was significantly associated with the illness. Subsequently, its leftovers were analyzed with polymerase chain reaction for the detection of *Cyclospora* DNA. The results were positive for the parasite’s presence and further sequencing analysis confirmed that the organism was *C. cayetanensis*. Raspberries were the only produce in the wedding cake and, therefore, the company’s raspberry distributors were interviewed to determine the source of the contaminated product. In addition, the FDA cooperated with the Guatemalan government and performed an inspection of a farm that was one of the raspberries’ suppliers to a bridal lunch held in Georgia in May 2000, which was also linked to a cyclosporiasis outbreak. Nevertheless, the FDA did not find an indisputable source of contamination.

This was the fifth outbreak of cyclosporiasis linked to Guatemalan raspberries which took place during spring in the United States and/or Canada and the first one linked to raspberries for which the parasite was detected in the epidemiologically implicated food. Since the 1996 and 1997 outbreaks, the Guatemalan government has cooperated with the FDA and the berry industry to set up standards and improve farming and exporting practices. These standards are updated on an annual basis. However, due to the outbreak which occurred in Pennsylvania and Georgia, the FDA prohibited the importation of raspberries from the Guatemalan farm that was common in both events in the spring of 2001 [43].

### 4.2. Basil

- United States, 1997

In July of 1997, state and local health departments in Virginia, Washington D.C. and Maryland were informed about cyclosporiasis cases that had occurred during June and July. On 7 July, the Alexandria Department of Health (ADOH) in Virginia was notified of a gastrointestinal illness developed by 54 employees who had attended a corporate luncheon on 26 June or had eaten leftover food on 27 June. The luncheon was catered by company A that operated in the northern Virginia-Washington D.C.-Baltimore, Maryland metropolitan area. Investigations demonstrated that basil-pesto pasta salad, produced and prepared by company A, was significantly associated with the illness. In addition to this, 185 cases (60 laboratory-confirmed and 125 clinically defined cases) and 75 clinically defined cases were reported to be associated with the events that occurred in the northern Virginia-D.C.-Baltimore metropolitan area during June and July. All 185 cases were caused by food consumption that contained fresh basil served from company A. At ADOH’s request, on 12 July, company A suspended sales of fresh basil and pesto sauce, as well as the production and sale of pesto sauce made with fresh basil and of food items containing this sauce. Additionally, on 18 July the health departments in Virginia and Maryland recommended that consumers should not eat fresh basil or food containing fresh basil purchased from company A [44]. The mode of contamination was not determined; however, this was the only cyclosporiasis outbreak with a notable possibility that basil was contaminated by a local US food handler [30].

- United States, 1999

On 11 and 23 August 1999, two clusters of cyclosporiasis were reported to the local health departments of Missouri. The clusters were linked to separate events both held on 24 July and caused the infection of 62 people. The first event was a birthday party which occurred in a country club and the second event was a graduation party in a restaurant. Of the 104 people attending the two events, 32 people reported illness after attending event A and 30 people reported illness after attending event B. In addition to these cases, three people who ate at the restaurant developed laboratory-confirmed cyclosporiasis.

Epidemiologic investigations of event A indicated that chicken pasta salad was strongly associated with the illness. The chicken pasta salad contained basil, red onions, and red bell peppers. However, only basil was likely to be associated with the illness. Epidemiologic investigations of event B showed that the tomato basil salad was the food most strongly associated with the outbreak. The tomato basil salad included basil, red onions, tomatoes, and shallots. Investigations performed to determine the source of the contaminated food indicated that the country club and the restaurant had a common basil distributor (distributor A). Distributor A received basil from distributor B who had previously purchased the item from a Mexican and a US farm. The research was not able to determine which farm supplied the basil that was finally served and how the contamination occurred. However, it was concluded that it was more likely that contamination occurred in the distribution chain.

This outbreak was the second reported outbreak of cyclosporiasis linked to the consumption of fresh basil and the first outbreak for which *Cyclospora* was isolated by an epidemiologically implicated food item [45].

- United States, 2019

As of 30 July 2019, 132 people with laboratory-confirmed cyclosporiasis were reported in 11 states (Connecticut, Florida, Georgia, Iowa, Massachusetts, Minnesota, New York, Ohio, Rhode Island, South Carolina, and Wisconsin). Four people were hospitalized, and no deaths were reported. Exposure occurred at restaurants in four states (Florida, Minnesota, New York, and Ohio) and the onset dates of illness ranged from 14 June to 9 July. Based on the CDC’s epidemiologic information consumption of contaminated fresh basil was the likely source of the outbreak, while based on the FDA’s traceback investigation the fresh basil was exported to the United States by Siga Logistics de RL de CV, located in Morelos, Mexico.

The firm voluntarily recalled the product; nevertheless, consumers were determined to avoid consuming basil or dishes garnished/ prepared with fresh basil (e.g., salads, fresh pesto) from Mexico if they did not know the exporting company or if they were not sure about its origin. Moreover, importers, suppliers, distributors, retailers, and other food service providers were advised not to sell, serve or distribute fresh imported basil if they did not know its source [46,47].

### 4.3. Snow Peas

- United States (Pennsylvania), 2004

The first documented outbreak of cyclosporiasis associated with the consumption of snow peas was reported in Pennsylvania, in 2004. During June and July, the public health authorities of Pennsylvania were informed about a cyclosporiasis infection which had developed among people in a residential facility. As of early July, 50 potential cases connected to the facility were reported to the local public authorities.

The CDC conducted stool examination and confirmed the presence of the parasite, while further investigations were performed to determine the source of the contamination. Of the 349 people associated with the facility, 315 were interviewed and 96 met the case definition (40 laboratory-confirmed cases and 56 probable cases). All cases were linked to five special events and, consequently, the investigation focused on detecting the common food item. Pasta salad was the only food strongly associated with the illness. The salad contained a variety of raw produce but only snow peas were linked to the outbreak. Traceback investigation indicated that all the snow peas used by the facility were from the same container, imported by a Guatemalan exporter. Nevertheless, by the time of the investigation, the product was not available for further analysis [48].

### 4.4. Cilantro

- United States (Iowa, Nebraska/Texas), 2013

In 2013, the FDA in coordination with US state and local public health officials started an investigation of a large number of reported cyclosporiasis cases, which occurred from June to August. As of 20 September, the CDC was informed of 643 cases from 25 states; most of the cases occurred in Texas (278), Iowa (153), and Nebraska (86).

Epidemiologic investigations indicated that most patients in Iowa and Nebraska developed illness from 15 June to 29 June, while Texas residents reported that the onset dates of symptoms were mainly from July to August. Furthermore, investigations in Iowa and Nebraska associated the illness with a mixed salad (iceberg lettuce, romaine lettuce, red cabbage, and carrots) served in restaurants. Traceback confirmed that a single lot of mixed salad supplied by one producer and distributor was associated with most of the confirmed cases [49]. In addition, the CDC, the FDA and state and local public health officials investigated a cluster of infections reported in a Mexican-style restaurant (restaurant A) in Fort Bend County, Texas. Case-control studies demonstrated that fresh cilantro was the only item significantly associated with the illness and traceback determined that it originated in Puebla, Mexico.

Combination of the investigation results indicated that lettuce, which was implicated with the illness in Iowa and Nebraska, was not associated with the outbreak in Texas; restaurant A did not use cabbage or carrots and the lettuce producer was not common. Hence, it was concluded that two unrelated cyclosporiasis outbreaks linked to lettuce and cilantro consumption had occurred in the US during summer 2013 [50].

### 4.5. Green Onions

- United States (Texas), 2017

In 2017, the Texas Department of State Health Services (DSHS) was informed of 20 cases of cyclosporiasis. Illnesses were reported between 21 July and 8 August by people who had dined at a Mediterranean-style restaurant chain in the Houston area. On 10 August, the CDC started an investigation conducted by the City of Houston Health Department, Harris County Public Health, Fort Bend County Health and Human Services, and Brazoria County Health Department to determine the source of infection.

Based on the case-control study, green onions were the only food item significantly associated with the illness. Traceback investigation was conducted to determine the source of the implicated food; however, the inspections that occurred in the outbreak-related restaurant failed to indicate the origin of the green onions [51].

### 4.6. Salads

- Germany, 2000

The first foodborne cyclosporiasis outbreak occurring in central Europe was reported in Germany in 2000. From 29 December 2000, until 18 January 2001, four clusters of gastroenteritis infection were reported to the German local health authorities. The clusters were independent and included 40 people who had attended luncheons in a common restaurant in southwest Germany on 13 and 14 December 2000. Patients’ stool samples were examined for intestinal protozoa in the department of a tropical medicine clinic and confirmatory tests performed at the State Health Office in Stuttgart and the Institute for General and Environmental Hygiene of the University of Tübingen confirmed the presence of *C. cayetanensis*. Based on the reports, illness developed in 34 people and the attack rate was 85%. Investigations indicated that two salads from side dishes were significantly associated with the illness and, therefore, the common components (lettuce varieties and fresh green leafy herbs) were reported to be the causative agents of the outbreak. However, no implicated food samples were available for further examination.

Traceback investigations showed that the butterhead lettuce batch had been grown in southern France and the mixed lettuce batch had been grown in the province of Bari in southern Italy. Regarding the fresh green leafy herbs, dill, parsley, and green onions were also grown in southern Italy (Naples, Eboli), while chives were the only ingredients grown in a greenhouse in Germany. Consequently, this outbreak was an alert to the authorities, highlighting the fact that cyclosporiasis in Europe could no longer be considered as exclusively travel related [52].

- United States, 2020

As of 12 August 2020, 690 patients were laboratory-confirmed with *Cyclospora* infection. Patients were residents in 13 states (Georgia, Illinois, Iowa, Kansas, Massachusetts, Minnesota, Missouri Nebraska, North Dakota, Ohio, Pennsylvania, South Dakota, and Wisconsin). The onset dates of the illness ranged from 11 May 2020 to 20 July 2020. Thirty-seven people were hospitalized and no deaths were reported. The age of the patients ranged from 10 to 92 years with a median age of 57 and 51% were female.

Epidemiologic and traceback investigations indicated that specific salad products made by Fresh Express were the likely source of this contamination. The contaminated bagged salad products were either branded with the Fresh Express brand or with the store brand labels ALDI Little Salad Bar, Giant Eagle, Hy-Vee, Jewel-Osco Signature Farms, ShopRite Wholesome Pantry and Walmart Marketside. Bagged salads were produced in the Fresh Express facility in Streamwood, Illinois, and were recalled by the company on the 27 June 2020. The contaminated salads contained lettuce, iceberg, red cabbage, and carrot and, even though they had best-before dates up to 14 July 2020, the CDC and the FDA continue their investigation to determine the affected ingredient in the salad mix and prevent other future product contaminations [53]. As of 14 August 2020, the FDA had investigated several farms and examined water samples from two public access points along a regional canal in Florida. Despite that water samples were tested positive for *C. cayetanensis,* there is currently not enough evidence to reveal the causative agent of this outbreak [54].

## 5. Travel Associated Cases

- Australia 2010

The first well described cyclosporiasis outbreak on a cruise ship was reported in 2010. This outbreak occurred in two successive cruises and was the first affecting a significant number of people in Australia. In June 2010, the Department of Health Western Australia (WA Health) was informed by a private laboratory that Cyclospora had been detected in five patients’ fecal samples. All patients reported having taken a cruise vacation that departed from Fremantle (Australia) on 14 May and returned on 31 May. During a subsequent cruise on the same ship, the ship’s medical clinic noticed an augmented number of gastrointestinal infections. A foodborne disease caused by a pathogenic bacterium was suspected and, therefore, ship’s staff informed the WA Health, which warned them that the infection might be due to *Cyclospora*. Stool samples were collected from 11 symptomatic passengers and analyzed when the ship returned to the port. The parasite was detected in 10 of them.

At least 314 people (266 passengers and 48 crew members) had a symptomatic infection, but the number of infected people was probably higher than this, since no systematic investigation was conducted. It was concluded that fresh produce purchased in Singapore or Malaysia was the causative agent of the outbreak. However, the exact item(s) that caused the illness was (were) not identified, since much fresh produce was consumed by patients during the cruise trip but no samples were collected for microbiological analysis [55].

- UK and Canada, 2015

From 1 June to 22 September 2015, 79 cases of cyclosporiasis (43 confirmed and 36 probable) were reported in England (55), Scotland (21), and Wales (3). Patients were interviewed about travel history, food consumption, clinical symptoms and demography, and results indicated that most of them had travelled to the Riviera Maya region of Mexico. The onset dates of illness ranged from 8 June to 19 August and based on the available information patients had travelled between 22 May and 30 August. The median age of the infected people was 44 years and 43 of them were female. No hospitalizations or deaths were reported. People had stayed in 32 different hotels in Riviera Maya, from Cancun to Tulum, and according to the 45 cases with available information, 43 had consumed berries, 41 salads or vegetables, and 35 fresh herbs.

At the same time, 97 cases of cyclosporiasis which developed in travelers returning from Mexico were reported in Canada. Ill people reported staying in various resorts in the same area as the UK patients. Investigations suggested that water was an unlikely source of contamination and that the outbreak was linked to a consumed food item(s) distributed through the area of Riviera Maya [56].

## 6. Control and Prevention

Cyclosporiasis is an infection that occurs after the consumption of contaminated foods or water, while person-to person transmission is unlikely. Therefore, avoiding food and water from contaminated sources is the optimum way to prevent *Cyclospora* infection, especially when water sanitation programs are less attentive (e.g., in developing countries). Good agricultural practices, such as rigorously sanitized irrigation water, can be conducive to decreased parasite contamination in the field and in the produce packing plants. Furthermore, good personal hygiene, appropriate food washing, and sanitary conditions can reduce the pathogen transmission, especially in endemic areas. However, it has been indicated that, even if these practices are strictly followed, the risk of exposure in this parasite may be reduced but not eliminated [4,77].

Hence, several studies have been performed to validate the efficiency of different hurdle methods in the reduction and inactivation of the parasite oocysts (viability indicator). According to Sathyanarayanan and Ortega [78], parasite’s oocyst exposure in extremely low or high temperatures (−70 °C, 70 °C and 100 °C) resulted in sporulation inactivation in basil samples, whereas room and refrigeration temperatures, did not affect the sporulation in various food samples. Treatment with chemical disinfectants has also been examined, with significant reductions in sporulation and protozoan population being reported. Magnesium oxide (MgO) nanoparticles showed antimicrobial activity against *C. cayetanensis* sporulated and unsporulated oocysts [79], while the population levels of the parasite decreased when fruits and vegetables were dipped in sodium dichloro-isocyanurate (NaDCC) solution (1 g/L) [80]. Despite this, chemical disinfection cannot guarantee product safety, since its efficacy depends on various factors, such as the initial population level of the microbe, the food matrix, and the environmental conditions [81]. In addition, irradiation (gamma and ultraviolet) and high-pressure technologies have also been evaluated utilizing surrogate parasites (e.g., *Eimeria acervulina*), revealing effective anti-parasite activities [82,83]. Nevertheless, the data are limited, and further research is necessary for the development of reliable control methods for this pathogenic parasite.

## 7. Discussion

Cyclosporiasis is a treatable gastrointestinal disease that predominantly occurs in tropical and subtropical countries. Infection occurs through the consumption of water or food contaminated with sporulated oocysts and has an average incubation period of 10 days. It is usually not life-threatening, but symptoms may persist for several weeks and can be severe, especially for high-risk population groups. Furthermore, it can result in long-term negative consequences when developed in young children, since illness might seriously affect their growth and cognitive development and even cause a chronic metabolic disease [84].

**Figure 1:**
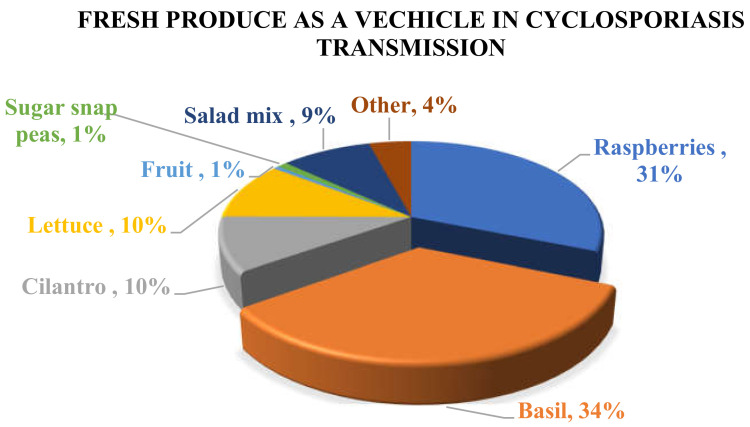
Distribution of *C. cayetanensis* in different fresh products that have been reported as the source of cyclosporiasis in humans from 1995–2019.

Until 1996, *Cyclospora* infection was rarely reported in non-endemic areas and most cases were thought to be associated only with international travel. However, the significantly increased rate of cyclosporiasis infection in non-endemic areas has changed the existing theory. In the USA and Canada, most of the reported cases have been linked to the consumption of produce imported from endemic countries, while in Europe and Australia cyclosporiasis outbreaks have been associated with international travel to endemic areas. Based on reported outbreaks and traceback and epidemiological investigations, basil and raspberries are the two main vehicles of Cyclospora infection in humans (Figure 1). Basil has been implicated in many cyclosporiasis outbreaks that have occurred mainly in the USA and Canada, while Guatemalan raspberries have also been indicated as the causative agent of many reported outbreaks. In addition, contaminated cilantro and lettuce have caused a significant number of cyclosporiasis infections worldwide (Table 1). In Europe, cyclosporiasis illness has been reported in Belgium, France, Germany, Greece, Ireland, Italy, The Netherlands, Spain, Sweden, and UK [4] but for most of the cases the vehicle of contamination was not determined, and the results of the investigations were not conclusive. It is also noteworthy that, even though the UK reported a significant number of cyclosporiasis outbreaks during 2015 (79), 2016 (440), and 2017 (58), only two other European countries reported a few cases of cyclosporiasis. In particular, France reported 9 cases (6 confirmed and 3 probable) in 2016 and Belgium reported 4 cases in 2017 [61].

The small number of reported cases in European countries could be attributed either to lack of travelers’ infection or underdiagnosis, under-reporting and lack of sufficient diagnostic capability [85]. *Cyclospora* diagnosis is often neglected and identification of the parasite is conducted only in patients with severe gastrointestinal symptoms, after several medical tests [61]. Furthermore, there are no validated molecular methods that can link illnesses to their sources at present, and therefore investigation of cyclosporiasis infections is mainly based on epidemiologic data [86]. In the USA, physicians have been asked to conduct laboratory tests for *Cyclospora* infection in patients with prolonged diarrhea with unidentified organisms [87]. Hence, cyclosporiasis outbreaks may remain undetected within the EU due to physicians’ failure to associated prolonged diarrhea with the specific infection.

## 8. Conclusions

*C. cayetanensis* was indicated as a foodborne pathogen in the mid-1990s. Since then, efforts have been made to identify the variability of the parasite and develop methods for its detection. However, the increase of foodborne outbreaks, especially related to produce consumption, continuously underlines the necessity for a better global understanding of the pathogen and the establishment of drastic control measures.

The low infectious dose and the long sporulation period complicate the performance of active surveillance. However, the outbreaks that occur highlight the necessity to overcome these difficulties and control the presence of the pathogen in water and fresh produce, especially in endemic countries. This will be achieved by cooperation between developed and developing countries. Developed countries ought to conduct further research on epidemiological aspects of this infection and coordinate with endemic countries to set up measures that will decrease the risk of producing and distributing unsafe food items.

At the same time, cyclosporiasis outbreaks that have occurred in developed countries have revealed the necessity to raise physicians’ awareness of this infection. Clinicians should be well informed about the infection’s typical symptoms and should request specific diagnostic tests for *Cyclospora* detection, especially in patients with prolonged diarrhea and travel history. Patients’ travel history should be recorded to provide important information for doctors and investigators and to assist investigators in determining the source of contamination. Moreover, clinical laboratories should be aware of the existing testing methods, and new techniques such as genome sequencing should be developed to investigate and detect the source of outbreaks.

Finally, it is noteworthy that the control procedures that currently exist in the fresh produce chain mainly examine the presence and levels of pathogenic and spoilage bacteria, without addressing the impact of these procedures on parasites’ survival. Hence, investigations on current methods and their effect on parasites, including *C. cayetanensis*, should be performed and addressed. In addition, novel methods that will ensure parasites’ control should be determined and included in the existing procedures that concern fresh produce safety.

## Figures and Tables

**Table 1:** Summary of additional cyclosporiasis outbreaks associated with fresh produce consumption reported from 1995 to 2019.

Date	Area	Number of Cases	Vehicle	Origin	Reference
1995 May	USA (Florida)	38	Raspberries ^a^	Guatemala ^a^	[30]
1995 May–June	USA (New York)	32	Fruit ^a^	UD ^b^	[30]
1997 May–June	USA (Florida)	305 ^a^	Mesclun ^a^	Peru or US ^a^	[30]
1997 September	USA (Virginia)	21	Fruit plate	UD	[30]
1997 April–June	USA Canada	1021	Raspberries	Guatemala	[57]
1998 May	USA (Georgia)	17	Fruit salad ^a^	UD	[30]
1999 May	USA (Florida)	94	Fruit/raspberries ^a^	UD	[30]
1999	Canada	104	Blackberries ^a^	Guatemala	[39]
2000 May	USA (Georgia)	19	Raspberries and/or Blackberries ^a^	Guatemala ^a^	[58]
2001 January–June	Canada (British Columbia)	30	Thai basil	UD	[59]
2001 April	Mexico (Monterrey)	97	Watercress	UD	[60]
2001 May	Canada (British Columbia)	17	Thai basil	Vietnam	[59]
2002 April	Colombia (Medellin)	56	Salads, juice	UD	[61]
2003 July	Canada (British Columbia)	11	Cilantro ^a^	UD	[62]
2003 May	Spain (Madrid)	13	Raspberry juice ^c^	Guatemala	[63]
2004 May–June	Canada (British Columbia)	8	Cilantro ^a^	UD	[62]
2004	Canada (British Columbia)	17	Mango or basil ^a^	UD	[62]
2005 April	Canada (Ontario)	44	Basil ^a^	Peru, Costa Rica	[62]
2005 April	United States (Florida)	592	Fresh basil ^a^	Peru	[64]
2005 June	Canada (Quebec)	250	Basil ^a^	Mexico	[62]
2005 June	USA (Connecticut)	30	Basil ^a^	UD	[58]
2006 June–July	Canada (British Columbia)	28	Basil or garlic	UD	[62]
2007 June	Canada (British Columbia)	23	Organic basil ^a^	Mexico	[65]
2008 March	USA (Wisconsin)	4	Sugar snap peas ^a^	Guatemala ^a^	[58]
2008	USA (California)	45	Raspberries and/or Blackberries ^a^	UD	[58]
2009 May–June	Sweden (Stockholm)	18	Sugar snap peas	Guatemala	[66]
2010 May	Canada (Ontario)	210	Fresh basil ^a^	UD	[67]
2013 June–August	USA (Texas)	270	Cilantro	Mexico	[68]
2013 July	USA (Wisconsin)	8	Berry salad ^a^	UD	[58]
2014 June–August	USA (25 states)	631	Romaine Lettuce	Mexico	[69]
2014 August	USA (19 states)	304	Fresh cilantro	Mexico (Puebla)	[70]
2015 May	USA (31 states)	546	Fresh cilantro	Mexico (Puebla)	[71]
2016 May–August	Canada (British Columbia, Alberta, Ontario, Quebec)	87	Fresh produce ^a^	UD	[72]
2018 May–June	USA (Minnesota, Wisconsin, Michigan, Iowa)	250	Vegetable trays ^d^	UD	[73]
2018 (May–July)	USA (16 states)	511	Salad mix	Illinois	[74]
2018 June	USA (2 states)	8	Basil	UD	[75]
2018 May–August	USA	53	Cilantro	Mexican-style restaurants	[75]
2019 May–August	USA (37 states, District of Columbia, and New York City)	2408	Basil	Mexico	[76]

^a^ Suspected; ^b^ Undetermined; ^c^ Travelers; ^d^ Pre-packaged vegetable trays containing broccoli, cauliflower, carrots, and dill dip.
